# IFNγ-producing iNKTs restrict a live-attenuated chlamydia oral vaccine in the large intestine

**DOI:** 10.3389/fimmu.2026.1851941

**Published:** 2026-06-01

**Authors:** Ahmed Mohamed Abdelsalam, Yi Wu, Mitchell Kronenberg, Huizhou Fan, Guangming Zhong

**Affiliations:** 1 Department of Microbiology, Immunology and Molecular Genetics, University of Texas Health Science Center at San Antonio, San Antonio, TX, United States; 2 La Jolla Institute for Immunology, La Jolla, CA, United States; 3 Department of Pharmacology, Robert Wood Johnson Medical School, Piscataway, NJ, United States

**Keywords:** chlamydia oral vaccine, IFNγ, iNKTs, intrOv, large intestine

## Abstract

*Chlamydia muridarum* continuously sheds live organisms and persists in the large intestine following intracolonic inoculation, while the live-attenuated chlamydia oral vaccine intrOv, an IFNγ-susceptible mutant of *C. muridarum*, fails to do so. IFNγ delivered by group 3 innate lymphoid cells (ILC3s) have been shown to block intrOv shedding. We now report that mice deficient in lymphocytes but competent in ILCs allowed intrOv to persist, revealing a critical role of lymphocytes for preventing intrOv persistence. The responsible lymphocyte subsets are natural killer T cells (NKTs), as mice deficient in either CD1d or β2m permitted intrOv to persist. We further narrowed the responsible cells to invariant NKTs (iNKTs) that produce IFNγ, as mice deficient in TCRα J18 segment (Traj18), T-bet, or IFNγ failed to prevent intrOv persistence. Consistently, intrOv induced IFNγ^+^iNKTs, and wild-type iNKTs prevented intrOv persistence in mice deficient in either lymphocytes or IFNγ. Thus, IFNγ^+^iNKTs are both necessary and sufficient for preventing intrOv persistence, while IFNγ^+^ILC3s are for blocking intrOv shedding, revealing a division of labor between IFNγ^+^iNKTs and IFNγ^+^ILC3s in regulating the interaction of the obligate intracellular Chlamydia with host mucosal tissue. The information is also essential for improving the safety and efficacy of intrOv as an oral vaccine.

## 1 Introduction

*Chlamydia* comprises multiple species of obligate intracellular bacteria that colonize mucosal tissues of different host species, with *Chlamydia trachomatis* infecting human mucosal tissues in the genital tract (serovars D to K) and the eye (serovars A to C), leading to severe complications (1–3). The genital *C. trachomatis* serovars are also frequently detected in the human gastrointestinal (GI) tract (4, 5). The mouse-adapted *C. muridarum* has been widely used to investigate the pathogenic mechanisms of *C. trachomatis* (6), leading to the discovery of a complex relationship between GI and genital *Chlamydia* (7–9). When an initial chlamydial infection in the genital tract ascends to the upper genital tract and spreads to the GI tract, GI chlamydia can persist for long periods (10), and promote the pathogenicity of genital chlamydia by inducing antigen-specific profibrotic CD8^+^ T cells (11, 12). On the contrary, if a naïve mouse is first exposed to chlamydia in the GI tract, GI chlamydia induces transmucosal protection against subsequent infections in extra-gut tissues (8, 9), which has motivated the development of live-attenuated chlamydia oral vaccines (13–15), one of which is designated as an intracellular oral vaccine vector, or intrOv.

Efforts to develop a human vaccine against chlamydial infections began with human trachoma vaccine trials more than 70 years ago (16–18). However, formalin-fixed whole chlamydial organism-based trachoma vaccines not only failed to induce long-lasting protection but also exacerbated ocular mucosal pathology. The failure had motivated the investigation of the pathogenic mechanisms of killed chlamydial organisms (19), and the development of subunit vaccines (20–24). While there is still no licensed human vaccine against *C. trachomatis*, the success in using modified *C. trachomatis* organisms to induce transmucosal protection in the female mouse genital tract (19) has rekindled efforts to develop whole-cell-based vaccines (25). Among the *C. muridarum* mutants that are attenuated in genital pathogenicity (26–31), some can induce transmucosal protection in the genital tract following oral inoculation (13–15). Since *C. muridarum* is known to induce cross-protection against *C. trachomatis* (32), an IFNγ-susceptible *C. muridarum* mutant clone designated intrOv is being developed as an oral vaccine to induce heterotypic transmucosal protection against *C. trachomatis* infection (33). To assess safety, intrOv was monitored in the GI tract following oral or intracolonic inoculation. Although wild-type *C. muridarum* continuously sheds live organisms and persists in the large intestine for long periods (34), intrOv is no longer able to persist in the large intestine and can only shed live organisms for a short period (35, 36). The IFNγ-producing group 3 innate lymphoid cells or IFNγ^+^ILC3s are responsible for preventing the shedding of live intrOv but without clearing its persistence in the large intestine (37–39). How the intrOv’s persistence is cleared remains to be addressed.

*Chlamydia* have been shown to interact with invariant natural killer T cells (iNKTs), although more studies are still needed to clarify the precise roles of iNKTs in chlamydial infection and pathogenicity (40–43). INKTs are a heterogeneous group of innate-like T cells (44). They express TCRs with limited diversity, with the α chain consisting of Vα14 and Jα18 in mice while human iNKTs use Vα24 and Jα18, which recognize glycolipids such as α-Galactosylceramide (αGalCer) as their ligand in the context of the CD1d antigen-presenting molecule complexed with β2M. INKT cells express transcription factors and cytokines similar to those expressed by subsets of CD4^+^ T cells, including T-bet and IFNγ (defined as NKT1), GATA3 and IL-13 (NKT2), and RORγt and IL-17 (NKT3). Since αGalCer-induced iNKTs were found to reduce chlamydial burdens in the lung (45), we hypothesize that iNKTs may also contribute to the inhibition of intrOv persistence in the GI tract by delivering IFNγ to large intestinal cells laden with intrOv.

The current study seeks to test the above hypothesis by identifying the immune components responsible for preventing intrOv persistence in the large intestine. We first compared intrOv tissue persistence patterns following intracolonic inoculation in mice with or without deficiency in all lymphoid cells (Rag2^-/-^γc^-/-^) or in conventional lymphocytes alone (Rag1^-/-^, or Rag2^-/-^). We found that intrOv continuously shed high levels of live organisms in rectal swabs and maintained high yields in tissues of mice deficient in all lymphoid cells (including ILCs and lymphocytes) but only maintained tissue persistence in mice deficient in lymphocytes. This finding validates the conclusion that ILCs can clear rectal shedding of intrOv but is insufficient to prevent intrOv’s tissue persistence, and it also suggests that lymphocytes are responsible for preventing intrOv persistence. To identify the responsible lymphocyte subsets, we compared intrOv persistence in mice deficient in specific immune components and found that inhibition of intrOv persistence depended on both CD1d and β2m, suggesting a critical role of NKTs. As intrOv persisted in mice deficient in the TCRα J18 segment (Traj18), T-bet, or IFNγ, the responsible cells are thus narrowed to IFNγ-producing iNKTs (IFNγ^+^iNKTs or NKT1). Consistently, we found that colonic intrOv induced a significant level of NKT1 cells, and adoptive transfer of wild-type iNKTs prevented intrOv persistence in mice deficient in either lymphocytes or IFNγ. Thus, IFNγ^+^iNKTs are both necessary and sufficient for preventing intrOv persistence, which, together with our previous findings on the clearance of intrOv shedding by IFNγ^+^ILC3s (37–39), reveals a clear division of labor between IFNγ^+^iNKTs and IFNγ^+^ILC3s in regulating the interaction between an obligate intracellular bacterium and mucosal tissue. The novel mechanistic information is also essential for improving the safety and efficacy of the live-attenuated oral vaccine intrOv.

## 2 Materials and methods

### 2.1 Chlamydial organisms

The *Chlamydia muridarum* wild-type clone G13.32.1 (WtCm) and mutant clone G28.51.1 designated an intracellular oral vaccine vector, or intrOv (6), were used in the current study. These clones are isogenic with each other (28, 29). IntrOv contains a glutamine (Q) to glutamic acid (E) substitutional mutation at the 117^th^ position of the hypothetical protein TC0237 (TC0237Q117E) and a deletion mutation in the *tc0668 gene*, converting the 216^th^ glycine (G) codon into a premature stop codon (TC0668G216*). The double mutations attenuate intrOv’s infectivity (46) and pathogenicity (29) in the genital tract and make intrOv susceptible to IFNγ (36, 37). However, intrOv still induces transmucosal protection in the genital tract following oral inoculation (13), while it is prevented from shedding live organisms from the GI tract by IFNγ-producing group 3 innate lymphoid cells or IFNγ^+^ILC3s (35–38, 47). Both WtCm and intrOv chlamydial organisms were grown in HeLa cells (human cervical carcinoma epithelial cells; ATCC# CCL-2), and a density-gradient purification protocol was used to purify the chlamydial elementary bodies (EBs) (48). The purified EBs were stored in aliquots at -80 °C until use.

### 2.2 Mouse infection and treatment

The mouse experiments were conducted in accordance with the recommendations outlined in the Guide for the Care and Use of Laboratory Animals, endorsed by the National Institutes of Health. The Protocol #20230086AR used in the current study was approved by the University of Texas Health San Antonio Institutional Animal Care and Use Committee (IACUC) under assurance ID D16-00224 (A3345-01). Prior to an intracolonic inoculation, mice were anesthetized with 3% isoflurane until they completely lost the righting reflex. After a quick intracolon inoculation, the mouse was placed on a warmed surface in a cage to recover before returning to the experimental cage. For non-survival surgeries, at the designated time-points or the conclusion of each animal experiment, mice were euthanized by overdose of isoflurane in a jar layered with paper towels and secured with a clear cover, followed by cervical dislocation, before collecting tissues and/or disposing of bodies. Prior to cervical dislocation, efforts were made to ensure that animals were unresponsive to toe stimulation.

The following 8- to 12-week-old male or female mice were used in the current study: C57BL/6J (C57, Jax stock# 000664, Jackson Laboratories, Inc., Bar Harbor, ME) as wild-type control mice, mice deficient in all lymphoid cells (lacking both recombinase Rag2 and IL-2 receptor common gamma chain, designated Rag2^-/-^γc^-/-^, Jax# 014593, C;129S4-*Rag2^tm1.1Flv^ Il2rg^tm1.1Flv^*/J), mice deficient in lymphocytes only (lacking recombinase Rag1, Rag1^-/-^, 002216, B6.129S7-*Rag1^tm1Mom^*/J or lacking recombinase Rag2, Rag2^-/-^, 008449, B6.Cg-*Rag2^tm1.1Cgn^*/J), mice deficient in MHCII (MHCII^-/-^, 003584, B6.129S2-*H2^dlAb1-Ea^*/J), mice deficient in β2m (β2m^-/-^, 002087, B6.129P2-*B2m^tm1Unc^*/DcrJ), mice deficient in CD1d [CD1d^-/-^, 008881, B6.129S6-Del(3Cd1d2-Cd1d1)1Sbp/Jor], mice deficient in TCRδ (TCRδ^-/-^, 002120, B6.129P2-*Tcrd^tm1Mom^*/J), mice deficient in CD19 (CD19^-/-^, 006785, B6.129P2(C)-*Cd19^tm1(cre)Cgn^*/J), mice deficient in TNFα (TNFα^-/-^, 005540, B6.129S-*Tnf^tm1Gkl^*/J), mice deficient in interleukin 17 A & F (IL-17A/F^-/-^, 034140, B6.Cg-*Il17a/Il17f^tm1.1Impr^ Thy1^a^*/J, but provided by Dr. Alexei Tumanov (University of Texas at San Antonio, San Antonio, TX), mice deficient in interleukin 22 (IL-22^-/-^, 027524, C57BL/6-*Il22^tm1.1(icre)Stck^*/J, Cre knock-in-knockout), mice deficient in IFNγ (IFNγ^-/-^, 017581, B6.129S4-Ifngtm3.1Lky/J, homozygous mice were used as knockouts), mice deficient in T-box transcription factor TBX21 (T-bet^-/-^, 004648, B6.129S6-Tbx21tm1Glm/J). Mice deficient in MR1 (MR1^-/-^) were generated by Dr. Susan Gilfillan (Washington University, St. Louis, MO) (49). Mice deficient in T cell receptor alpha chain J18 segment (Traj18^-/-^) were from Dr. Kronenberg (La Jolla Institute for Immunology, La Jolla, CA) (50). It is worth noting that the Traj18^-^/^-^ mice used in the current study do not perturb the broader Jα repertoire (unlike the original Traj18 knockout, which suppresses upstream Jα rearrangements), allowing us to validate the specific dependence of the observed phenotypes on iNKTs.

All mice were inoculated intracolonically without or with WtCm and intrOv EBs at a dose of 1 x 10^5^ and 1 × 10^7^ inclusion forming units (IFUs) per mouse, respectively, as described previously (13, 38, 51). Briefly, EBs diluted in 50μl of SPG buffer (220 mM sucrose, 12.5 mM phosphate, 4 mM l-glutamic acid, pH 7.5) were delivered to the colon using a straight ball-tipped needle (N-PK 020; Braintree Scientific, Inc., Braintree, MA) following anesthesia. After inoculation, mice were monitored for live chlamydial organisms in rectal swabs or sacrificed for titrating live organisms in organs/tissues, including small intestine (SI), various large intestine (LI) segments such as cecum (Cec), colon (Col), anorectum (AR), and extra gastrointestinal (GI) organs such as spleen (Spl), liver (liv), & kidney (Kid).

### 2.3 Preparing swab and tissue samples for titrating live chlamydial organisms

Rectal swabs were used to monitor live chlamydial organism shedding from the GI tract, while live chlamydial organisms were recovered directly from individual GI tract tissues to monitor chlamydial persistence. Each rectal swab was collected in 0.5 ml of SPG buffer and vortexed with glass beads to release infectious chlamydial EBs. To directly recover live chlamydial organisms from individual tissues/organs, tissues/organs were collected on the designated days, as indicated in each experiment, into 2 ml of SPG buffer per tissue (5 ml SPG for SI). Each tissue was homogenized and sonicated to release live chlamydial organisms into the solution. After a brief centrifugation to pellet cell debris, the live chlamydial organisms in the supernatant were titrated in duplicate on HeLa cell monolayers grown in 96-well plates. A total volume of 100μl of each sample, without (neat) or with serial dilutions, was used to inoculate monolayer HeLa cells. After incubation at 37 °C, the infected HeLa cell monolayers were processed for immunofluorescence labeling of chlamydial organisms as described below.

### 2.4 Immunofluorescence assay and enumeration of live chlamydial organisms in each sample

An immunofluorescence assay described previously (52) was used to visualize chlamydial inclusions. Briefly, infected HeLa cells grown on 96-well plates were fixed with paraformaldehyde (Sigma, St. Louis, MO) and permeabilized with Triton X-100 (Sigma). The monolayers were labeled with a rabbit anti-chlamydial antibody (raised by immunization with *C. muridarum* EBs), and a goat anti-rabbit IgG conjugated with Cy2 (green; Jackson ImmunoResearch Laboratories, Inc, West Grove, PA) to visualize chlamydial inclusions, while a Hoechst dye (blue; Sigma) was used to label nuclear DNA. The labeled cell samples were viewed under an Olympus IX-80 fluorescence microscope equipped with multiple filter sets (Olympus, Melville, NY).

Five random views per well were counted, or the entire well was counted for chlamydial inclusions when the inclusion density was low, allowing the detection of a single IFU per 100μl sample. This practice should ensure the detection sensitivity of the assay at 20 IFUs per sample. The total number of IFUs per swab or tissue was calculated based on the # of IFUs per well, the inoculum volume, and sample dilutions, and the results were converted to log_10_ IFUs for group comparisons.

### 2.5 Lymphoid cell isolation from mouse tissues

Lymphoid cells were prepared from mouse tissues as described previously (38, 53). For iNKT cell induction and isolation, each donor mouse was injected intraperitoneally with 10 µg/ml αGalCer (KRN7000, Hello Bio Inc, Princeton, NJ) in 200 µL PBS. Five days later, a single-cell suspension was obtained from the spleen and liver tissues harvested from euthanized mice (54, 55). Both the spleen and liver were gently homogenized using the back of a sterile syringe plunger, filtered through a 70 μm strainer, and incubated in Ammonium-Chloride-Potassium lysis buffer (A1049201, Thermo Fisher Scientific, Inc, Waltham, MA) for 10 min on ice, followed by two washes with 1X PBS. The spleen single-cell suspension was kept on ice, while the liver cell suspension underwent Percoll purification (**Supplementary Figure 1**). Subsequently, both purified single-cell suspensions were stained for sorting.

To detect NKT1, mice were inoculated intracolonically with 1 x 10^7^ IFUs of intrOv or SPG. Seven days after Inoculation, the small and large intestinal tissues, and the liver were excised for lymphoid cell isolation. Liver lymphoid single cell suspension was obtained as described above, while the small intestine and large intestine were minced into 1–2 mm pieces. The minced tissue mixture was digested with DNase I and collagenase II, then shaken at 37˚C for 50 min to release lymphoid cells into the supernatant. The process was repeated using both EDTA and DTT for 20 min. After filtering and washing, the lymphoid cell-containing solutions were collected for Percoll purification. The lymphoid cell-containing interphase was harvested as lymphoid cells for flow cytometry analysis (**Supplementary Figure 2**).

### 2.6 Flow cytometry analysis and sorting

CD1d-αGalCer tetramer specificity was confirmed by preparing single-cell suspensions from the liver of C57 mice and Traj18-/- mice. The cells were labeled with a rat anti-mouse CD16/32 antibody (to block nonspecific binding to Fc receptors, clone: 2.4G2, cat#: BE0307, Bio X Cell, West Lebanon, NH), rat anti-mouse CD45 antibody conjugated with BUV737 (clone# 30-F11, cat#: 367-0451-82, Thermo Fisher Scientific), the viability dye (eFluor 506, cat#: 65-0866-14), anti-CD3ε antibody conjugated with PE-CY7 (clone: 17A2, cat#: 25-0032-82), CD1d-αGalCer tetramer conjugated with BV421 (cat#: MCD1d-001, Tetramerstore, Denmark), and CD1d empty tetramer conjugated with APC-CY7 (cat#: MCD1d-002, Tetramerstore, Denmark) (**Supplementary Figure 3**).

For flow cytometry analyses, the following antibodies were used to label cell surface markers: Rat anti-mouse CD16/32, rat anti-mouse CD45, viability-dye, anti-NK1.1, anti-TCRβ, and CD1d-αGalCer tetramer. All labeling was performed for 30 min at 4°C. After washing, the surface-labeled cells were fixed and permeabilized for intracellular labeling with rat anti-mouse IFNγ antibody conjugated to FITC (clone XMG1.2, cat#11-7311-82, Thermo Fisher Scientific). The Cytek Aurora instrument was used to analyze the antibody-bound samples.

To sort-purify iNKTs (TCRβ+NK1.1+CD1d-αGalCer tetramer+) and non-iNKTs (TCRβ+NK1.1+CD1d-αGalCer tetramer-) as donor cells for adoptive transfer, lymphoid cells isolated from mouse tissues were labeled with viability dye and antibodies against CD45, NK1.1, and TCRβ, respectively, as well as CD1d-αGalCer tetramer. After gating for live CD45+NK1.1+, cells were sorted for TCRβ+ and CD1d-αGalCer tetramer+ as iNKTs or TCRβ+ and CD1d-αGalCer tetramer-as non-iNKTs using BD FACSDiscover™ S8 Cell Sorter, BD Biosciences (**Supplementary Figure 4**).

### 2.7 Adoptive transfer

Donor lymphoid cells prepared from C57 mice as described above were injected retro-orbitally into recipient mice at a dose of 5 x 10^4^ each. The transfer was carried out, once, 24 hr. prior to the intracolonic inoculation of intrOv. Following intrOv inoculation, live intrOv organisms were monitored in both rectal swabs and tissues to assess the impact of donor cells on intrOv colonization in recipient mice.

### 2.8 Statistics

The number of live chlamydial organisms per sample (expressed as log10 IFUs/sample) was compared at individual data points or over a time course (area under the curve or AUC) using the Wilcoxon rank-sum test. When multiple groups were included, ANOVA was first used to assess whether there was a significant difference among them. Only when p<0.05 (ANOVA) were the differences between the two given groups further analyzed. For these pairwise comparisons, p-values were adjusted for multiple testing using the Benjamini-Hochberg method.

## 3 Results

### 3.1 Rectal shedding of the live-attenuated chlamydia oral vaccine intrOv is blocked by innate lymphoid cells, while its tissue persistence is prevented by lymphocytes

**Figure 1:**
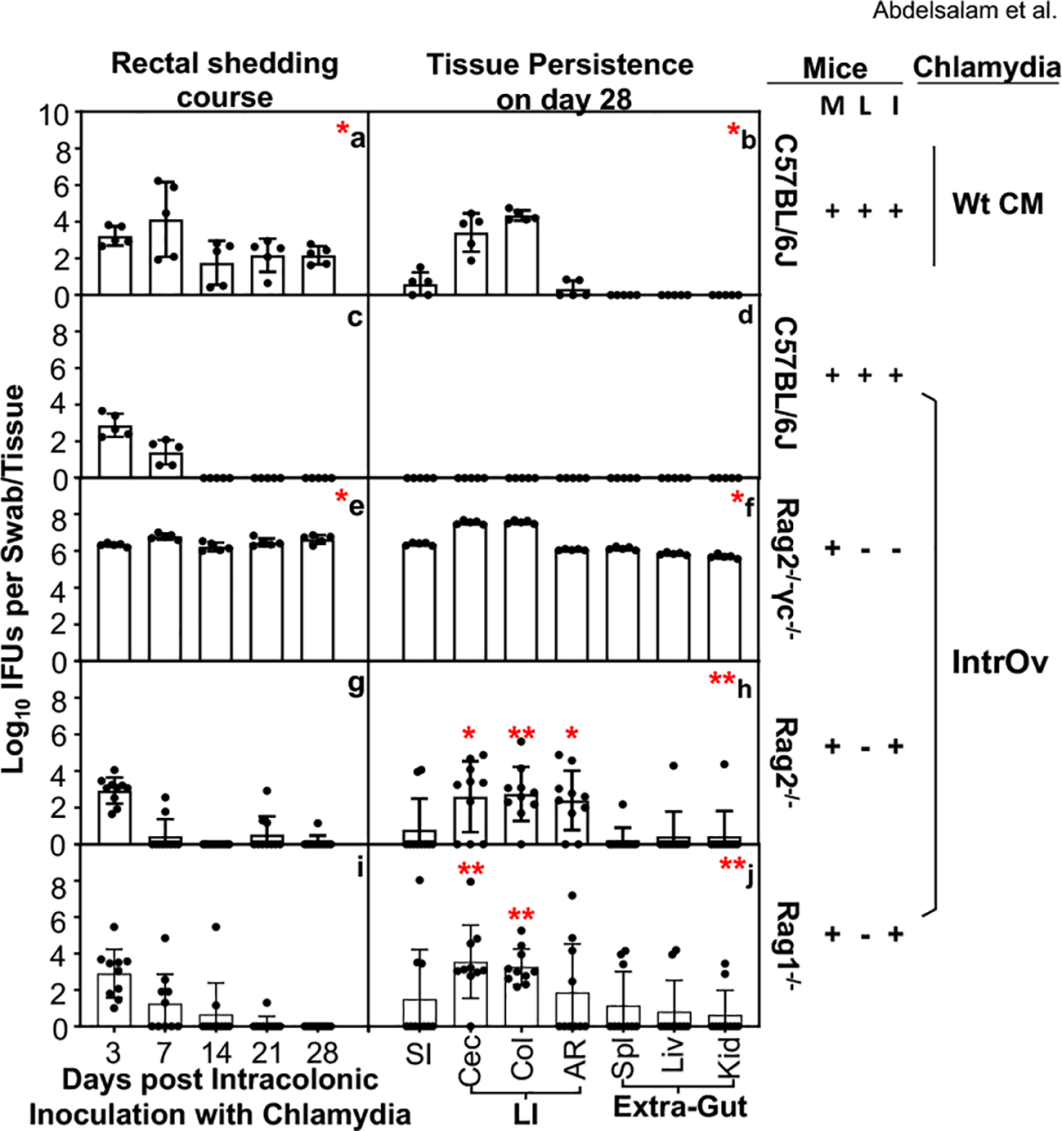
Comparison of rectal shedding and tissue persistence of the live-attenuated oral vaccine intrOv in mice deficient in conventional lymphocytes alone versus all lymphoid cells. Mice without (C57BL/6J, panels **(A–D)**, n=5) or with a deficiency in all lymphoid cells (Rag2^-/-^γc^-/-^, e & f, n=5) or conventional lymphocytes only (Rag2^-/-^, g & h, n=10, or Rag1^-/-^, i & j, n=10) were inoculated intracolonically with wild-type *Chlamydia muridarum* (Wt CM, **(A, B)**) at the dose of 1 x 10^5^ IFUs (inclusion forming units) or a live-attenuated oral vaccine strain (IntrOv, **(C–J)**) at a dose of 1 x 10^7^. All mice were monitored for live chlamydial loads in rectal swabs taken on days 3, 7, and weekly thereafter **(A, C, E, G, I)**, and in tissues harvested on day 28 **(B, D, F, H, J)**. The tissues include the small intestine (SI), the large intestine (LI) segments such as cecum (Cec), colon (Col), rectum (AR), and extra-gut organs such as spleen (Spl), liver (Liv), and kidney (Kid). The recovered chlamydial loads were expressed as log10 IFUs per swab or tissue (along the left Y-axis). The presence or absence of myeloid (M), conventional lymphocytes (L), or innate lymphoid cells (I) from each strain of mice are indicated on the right. *P<0.05 whereas **P < 0.01, Wilcoxon rank sum, AUCs (area-under curves) were compared: panel **(A)** vs. **(C)** and **(B)** vs. **(D)**, **(C)** vs. **(E, G)**, or **(I, D)** vs. **(F, H, J)**, while IFUs at different time points or from individual tissues were compared between panel **(C)** vs. **(G I)**, and between **(D)** vs. **(H, J)** Data were from 3 independent experiments. Note that while IntrOv shed live organisms in the rectal swabs and persisted in all tissues of mice lacking all lymphoid cells, it could only persist in the large intestine of mice lacking conventional lymphocytes.

To identify the immune responses responsible for regulating colonization of the live-attenuated chlamydia oral vaccine intrOv in the large intestine, we compared the rectal shedding and tissue persistence of intrOv following intracolonic inoculation in mice with or without deficiency in all lymphoid cells (Rag2^-/-^γc^-/-^) or in conventional lymphocytes alone (Rag2^-/-^ or Rag1^-/-^). As shown in **Figure 1**, Rag2^-/-^γc^-/-^ mice continuously shed high levels of intrOv in the rectal swabs throughout the course of observation and maintained high yields from all tissues examined on day 28, while in wild-type C57 mice, intrOv was rapidly cleared from rectal shedding and effectively prevented from persistence in any tissue. These observations are consistent with our previous finding that intrOv shedding in the rectum is rapidly cleared by IFNγ^+^ILC3s (36, 37). However, in mice lacking conventional lymphocytes due to a deficiency in recombinase Rag1 or Rag2, which is required for the formation of lymphocyte antigen receptors, intracolonically inoculated intrOv failed to maintain significant shedding but still persisted at a significantly higher level in large intestinal tissue on day 28 compared with C57 mice. These results demonstrate that Rag1 or Rag2-dependent lymphocytes are required to prevent intrOv from persisting in the large intestine, suggesting a division of labor between Rag-independent innate lymphoid cells and Rag-dependent lymphocytes in inhibiting intrOv shedding and preventing intrOv tissue persistence, respectively.

### 3.2 Invariant natural killer T cells are responsible for preventing intrOv persistence in the large intestine

**Figure 2:**
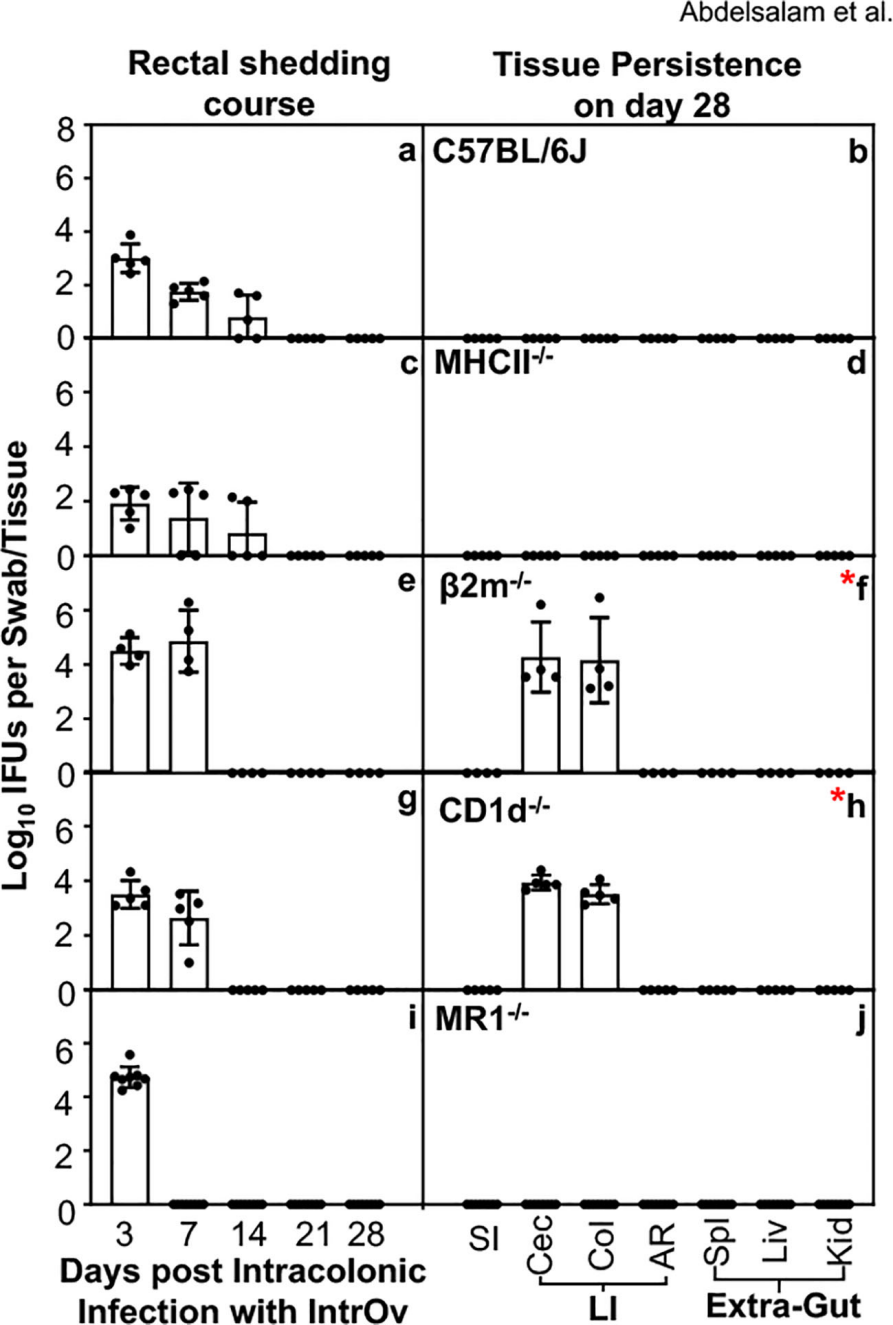
Comparison of intrOv shedding and persistence in mice deficient in different antigen-presenting molecules. Groups of mice without (C57BL/6J, panels **(A, B)**, n=5), or with deficiency in MHCII (MHCII^-/-^, **(C, D)**, n=5), β2m (β2m^-/-^, **(E, F)**, n=5), CD1d (CD1d^-/-^, **(G, H)**, or MR1 (MR1^-/-^, **(I, J)**, n=5) were inoculated with IntrOv and monitored for rectal shedding in the rectal swabs **(A, C, E, G, I)** and bacterial loads from different organs/tissue segments **(B, D, F, H, J)** as described in the legend of **Figure 1** above. The results, expressed as log10 IFUs, were compared between the C57BL/6J group and the other groups at the different time points/tissue types and overall AUCs as described in the legend of **Figure 1**. *P<0.05, Wilcoxon rank sum (2-tailed). Data were from 2 independent experiments. Note that both β2m and CD1d are required for inhibiting IntrOv persistence in the large intestine.

**Figure 3:**
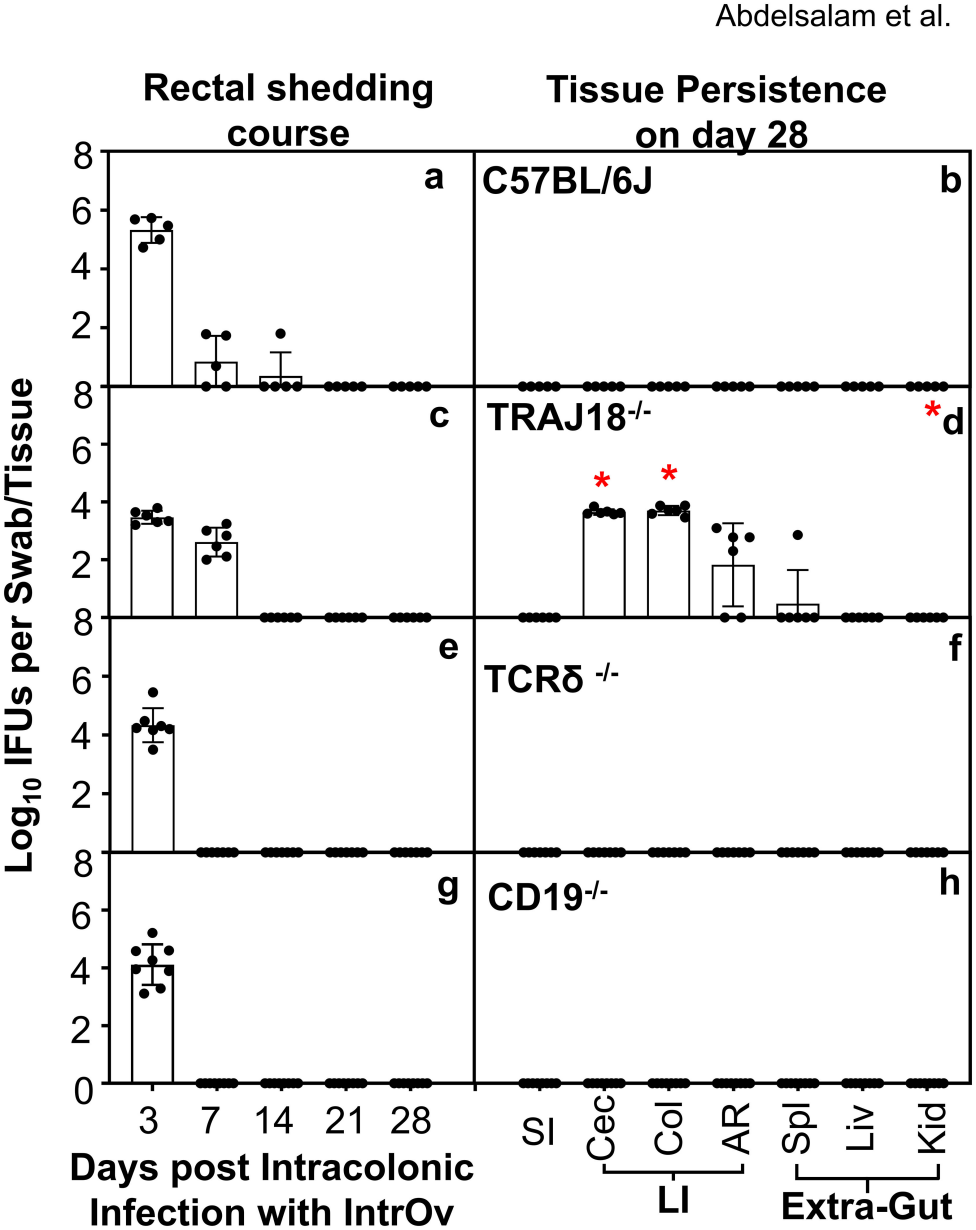
Comparison of intrOv rectal shedding and tissue persistence in mice deficient in different subsets of lymphocytes. Wild type C57BL/6J mice (panels **(A, B)**, n=5), or mice deficient in TRAJ18 (TRAJ18^-/-^, **(C, D)**, n=5), TCRδ (TCRδ^-/-^, **(E, F)**, n=7), or CD19 (CD19^-/-^, **(G, H)**, n=8) were intracolonically inoculated with IntrOv and monitored for live chlamydial loads in rectal swabs **(A, C, E, G)** and in tissues **(B, D, F, H)** as described in the legend of **Figure 1**. The chlamydial load results were expressed as log10 IFUs as displayed along Y-axis and compared between the C57BL/6J group and the other groups. *P<0.05, Wilcoxon rank sum (two-tailed). Data were from 2 or 3 independent experiments. Note that IntrOv persists in mice deficient in TRAJ18 but not the other immunodeficient mice.

To identify the lymphocyte subsets responsible for preventing intrOv from persisting in the large intestinal tissue, we first compared mice with or without deficiencies in molecules required for antigen presentation to different T lymphocyte subsets (**Figure 2**). Just like the wild-type C57 mice, mice deficient in MHCII (MHCII^-/-^) or MR1 (MR1^-/-^) both rapidly cleared the rectal shedding and fully prevented tissue persistence of intrOv, suggesting that neither conventional CD4^+^ T cells nor the mucosa-associated invariant T cells (MAITs) are required for preventing intrOv persistence. In contrast, mice deficient in β2m (β2m^-/-^) or CD1d (CD1d^-/-^) failed to prevent intrOv from persisting in the large intestinal tissues (cecum and colon), although these mice rapidly cleared rectal shedding of intrOv as efficiently as wild-type mice. These results suggest that natural killer T cells (NKTs) are responsible for preventing intrOv’s tissue persistence, as CD1d in association with β2m is required for presenting glycolipid antigens to NKTs. To support this conclusion, we further compared mice with or without deficiencies in different lymphocyte receptors (**Figure 3**). Mice deficient in either TCRδ (TCRδ^-/-^) or CD19 (CD19^-/-^) both rapidly cleared the rectal shedding and prevented tissue persistence of intrOv, confirming that neither gamma/delta T cells nor B cells are required for controlling intrOv colonization in the large intestine. However, mice deficient in the T cell receptor alpha chain J18 segment (Traj18^-/-^) allowed intrOv to persist for a significant period in the large intestinal tissues, including the cecum and colon, as detected on day 28 post-inoculation. This observation not only validates the above conclusion but also narrows the responsible cells to invariant NKT cells.

### 3.3 IFNγ-producing iNKT or NKT1 cells are sufficient to clear intrOv persistence in the large intestine.

**Figure 4:**
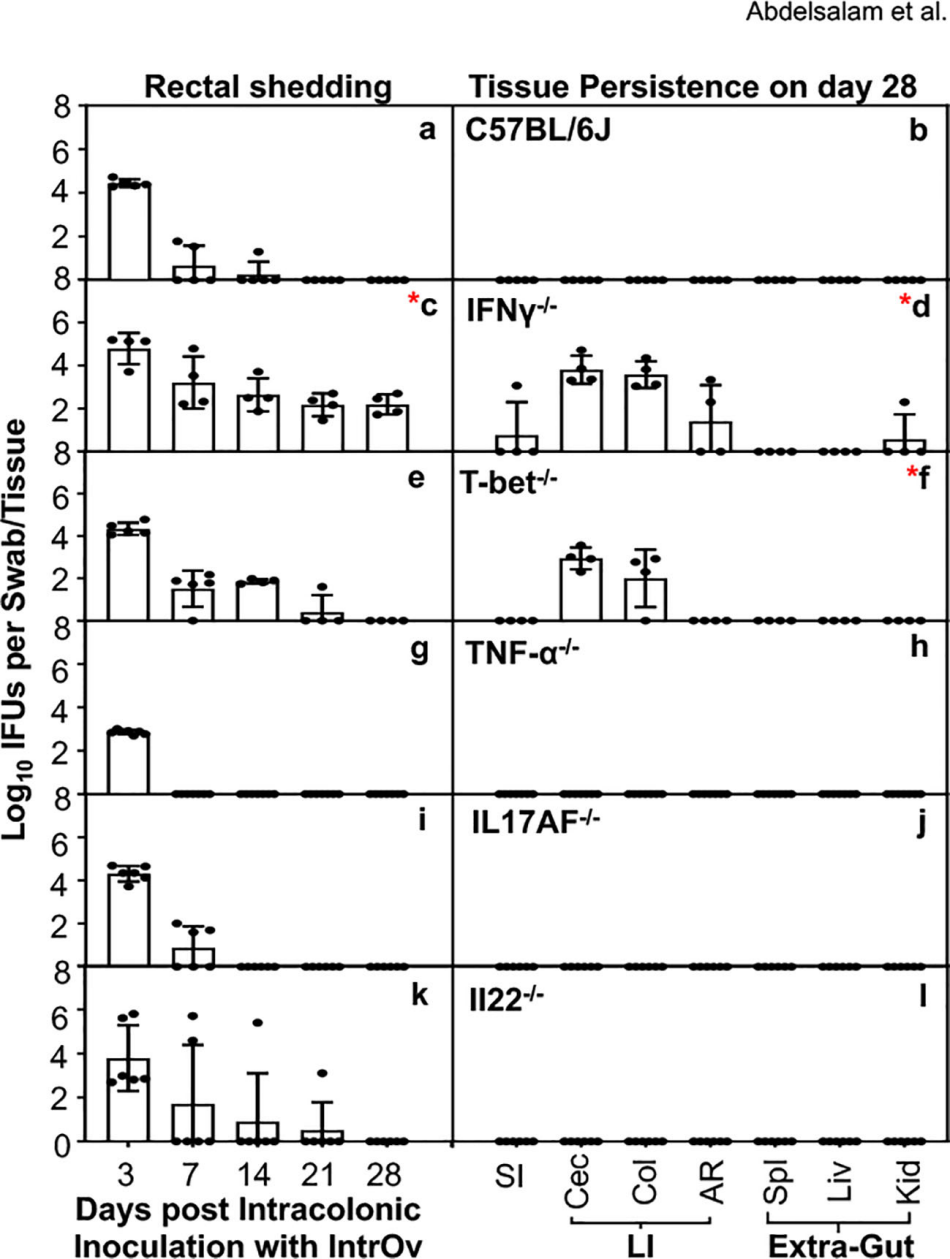
Comparison of intrOv rectal shedding and tissue persistence in mice deficient in cytokines or transcriptional factors. Wild type C57BL/6J mice (panels **(A–D)**, n=5), or mice deficient in interferon gamma (IFNγ^-/-^, **(C, D)**, n=4), T-box transcription factor TBX21 (T-bet^-/-^, **(E, F)**, n=4), tumor necrosis factor alpha (TNFα^-/-^, **(G, H)**, n=7), interleukin 17 A & F (IL-17A/F^-/-^, **(I, J)**, n=6), or interleukin 22 (IL-22^-/-^, **(K, L)**, n=6) were intracolonically inoculated with IntrOv and monitored for live chlamydial loads in rectal swabs **(A, C, E, G)**, and in tissues **(B, D, F, H)** as described in **Figure 1**. legend. The chlamydial load results were expressed as log10 IFUs as displayed along the left Y-axis and compared between C57BL/6J group and each other group. *P<0.05, Wilcoxon rank sum (two-tailed). Data were from 2 or 3 independent experiments. Note IntrOv persists in mice lacking IFNγ or T-bet.

**Figure 5:**
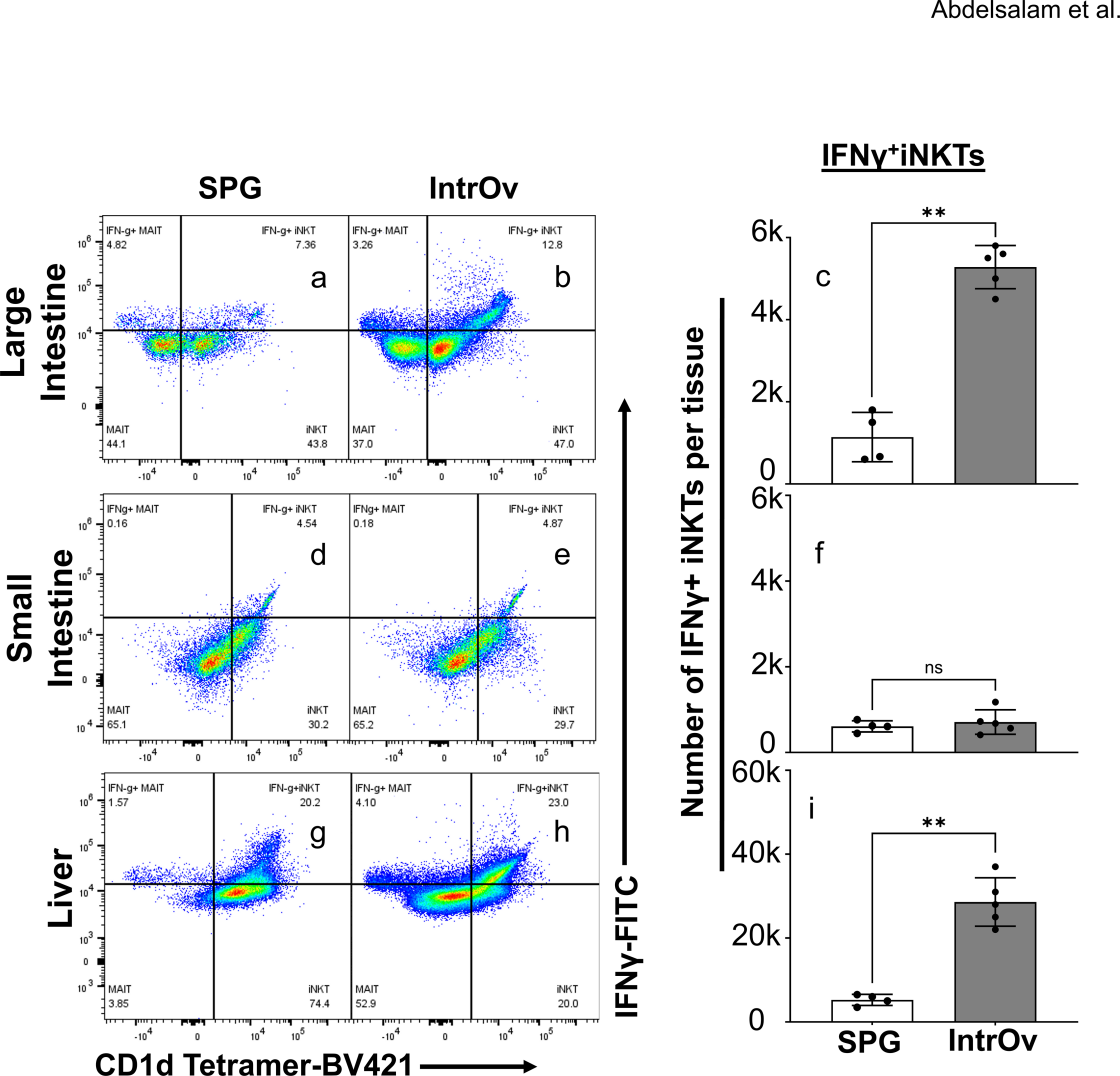
Induction of IFNγ-producing iNKTs by colonic intrOv. Wild type C57BL/6J mice inoculated with 50µl of sucrose phosphate glutamate solution alone (SPG, panels **(A, D, G)**, n=4), or with 50µl SPG containing 1 x 10^7^ IFUs of IntrOv (**(B, E, H)**, n=5). On day 7 post-inoculation, the large intestine **(A–C)**, small intestine **(D–F)**, or liver **(G–I)** organs were harvested for single-cell suspensions. After gating for single live cells, CD45^+^, NK1.1^+^, & TCRβ^+^, the above-gated cells were further dually labeled using CD1d-αGalCer tetramer and anti-IFNγ to identify IFNγ-expressing αβiNKTs from the large intestine **(A, B)**, small intestine **(D, E)**, and liver **(G, H)** and the double positive cells from each organ were summarized in panels c (large intestine, open bar for cells from SPG-treated mice while gray bar for cells from IntrOv-treated mice), **(F)** (small intestine), and **(I)** (liver), respectively. **P < 0.01, ns denotes not significant, Wilcoxon rank sum (two-tailed). Data were from 3 independent experiments. Note that colonic intrOv significantly increases IFNγ+αβiNKTs in both the large intestine and liver, but not the small intestine.

**Figure 6:**
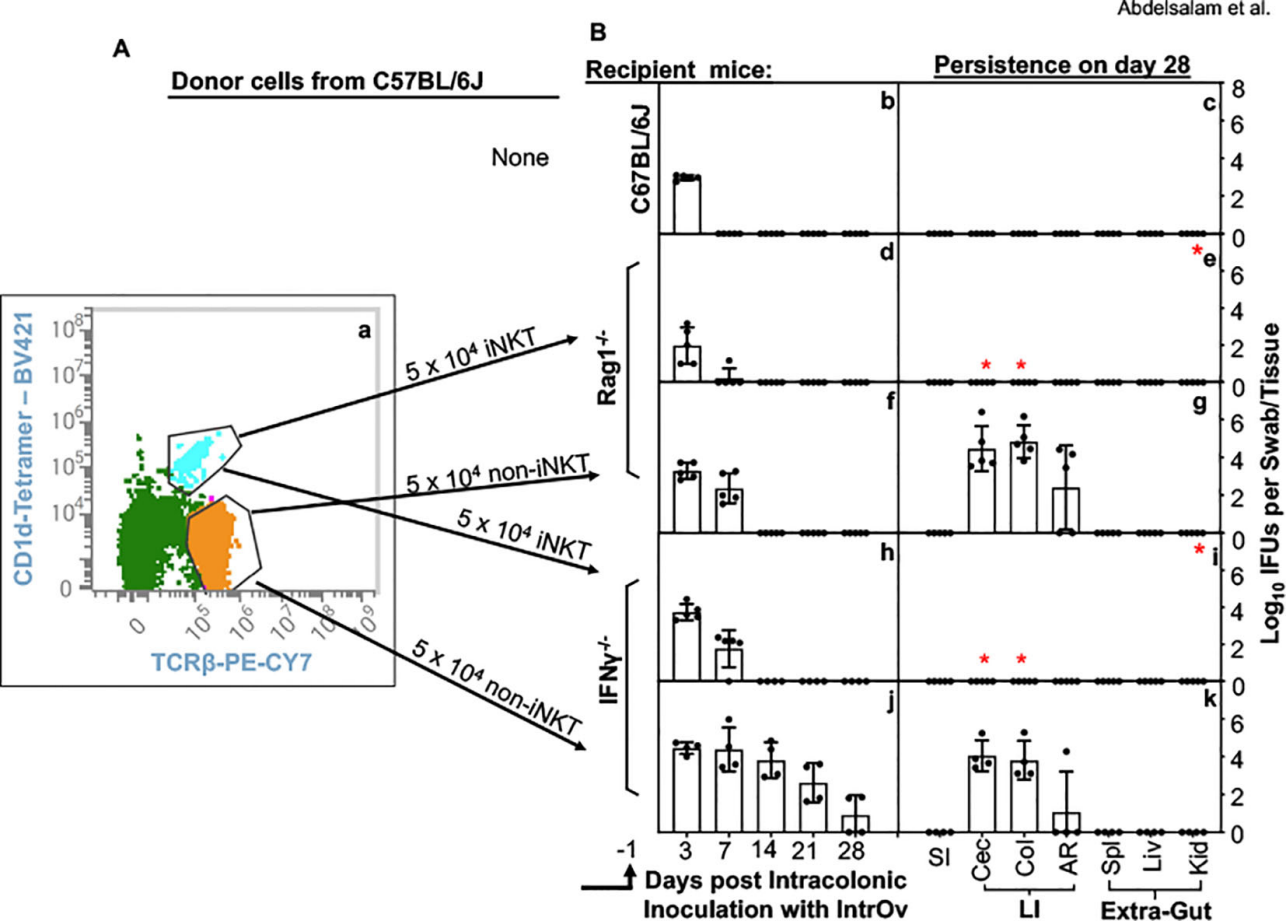
Adoptive transfer of wild-type iNKTs to rescue the inhibition of intrOv persistence in mice lacking conventional lymphocytes or IFNγ. **(A)** C57BL/6J mice were induced to produce iNKTs using αGalCer, and CD45+ cells were harvested from the mouse liver and sorted as αβiNKTs or αβMAITs as donor cells (panel (a)) for the adoptive transfer experiment. **(B)** The C57BL/6J mice without receiving any donor cells were used as the control recipient mice that restrict IntrOv colonization. Mice deficient in conventional lymphocytes (Rag1^-/-^, panels (d–g)) or IFNg (IFNg^-/-^, (h–k)) were used as recipient mice for receiving 5 x 10^4^ of sorted donor cells. The sorter-purified αβiNKTs (CD45+Nk1.1+TCRβ+αGalCer loaded CD1d-Tetramer+) were transferred to Rag1^-/-^ (panels (d, e), n=5) or IFNγ ^-/-^ ((h, i), n=4) mice via retro-orbital injection. The sorter-purified αβMAITs (CD45+Nk1.1+TCRβ+αGalCer-loaded CD1d-Tetramer-) were similarly transferred to the recipient mice Rag1^-/-^ (panels (f, g), n=5), or IFNγ ^-/-^ (j, k), n=4). Twenty-four hours after the adoptive transfer, the recipient mice were intracolonically challenged with 1 x 10^7^ IFUs of IntrOv and then monitored for live chlamydial organism shedding in vaginal swabs (a, d, f, h, j) and yields from mouse tissues as described in **Figure 1** legend. The results, expressed as log10 IFUs, were compared between recipient mice receiving donor cells αβiNKTs versus αβMAITs. *p<0.05, Wilcoxon rank-sum (two-tailed). Data were from 2 or 3 independent experiments. Note that αβiNKTs inhibit IntrOv persistence in both Rag1^-/-^ and IFNg^-/-^ mice.

To understand how iNKTs prevent the tissue persistence of intrOv, we compared mice with or without deficiencies in different cytokines and transcriptional factors (**Figure 4**). Mice deficient in tumor necrosis factor alpha (TNFα^-/-^), interleukin 17 A & F (IL-17A/F^-/-^), or interleukin 22 (IL-22^-/-^) all prevented intrOv persistence as efficiently as the wild-type C57 mice, suggesting that these cytokines are not required for the iNKTs to prevent the tissue persistence of intrOv. However, mice deficient in interferon gamma (IFNγ^-/-^) or T-box transcription factor TBX21 (T-bet^-/-^) permitted intrOv to maintain significant persistence in the large intestine, suggesting that the responsible iNKTs may use the T-bet-dependent IFNγ production to inhibit intrOv’s persistence, although more experiments are required to directly tie the T-bet dependence to iNKTs’ ability to express IFNγ. Consistently, intracolonically inoculated intrOv induced significant levels of IFNγ-producing iNKTs (IFNγ^+^iNKTs) in both the large intestine and the liver, but not in the small intestine, on day 7 post-inoculation (**Figure 5**), demonstrating the availability of IFNγ^+^iNKTs in the large intestine for inhibiting intrOv persistence (Gating strategy, Extended **Figure 5**). To further test whether these iNKTs are sufficient to prevent intrOv from persisting in the large intestine, we used an adoptive transfer approach (**Figure 6**). We found that adoptive transfer of wild-type iNKTs rescued the inhibition of intrOv persistence in the large intestine of either Rag1^-/-^ or IFNγ^-/-^ recipient mice. A similar transfer of non-iNKT cells, sorted from the same wild-type donor mice, failed to inhibit intrOv persistence in recipient mice, suggesting that donor cell-dependent inhibition of intrOv persistence is specific to iNKTs.

## 4 Discussion

Despite extensive efforts over the past 70 years to develop a human vaccine to prevent *C. trachomatis* infection, no vaccine is currently licensed. The *C. muridarum*-based intrOv is being developed as a live-attenuated oral vaccine to protect humans against *C. trachomatis* infection in the genital tract (33), supported by findings that oral administration of intrOv induced transmucosal protection in the genital tract of mice (13) and that *C. muridarum* elicits heterotypic protection against *C. trachomatis* (32). Despite its potent immunogenicity, intrOv appears highly attenuated in both the genital (29, 46) and GI tracts (35, 56). However, the mechanisms of intrOv’s attenuation remain unknown. Revealing the immune mechanisms induced by intrOv to both clear the attenuated intrOv itself and prevent subsequent infection by wild-type chlamydia should provide essential information to improve the safety and efficacy of intrOv and design future oral vaccines. We have used an intracolonic inoculation mouse model to investigate these mechanisms because it is simpler, and the rapid clearance of the intracolonically inoculated intrOv from wild-type mice provides a convenient readout for monitoring the immune responses responsible. For example, the intracolonic model has already allowed us to identify IFNγ^+^ILC3s as responsible for preventing intrOv from shedding live organisms (36–38). However, the immune mechanism responsible for blocking intrOv from persisting in tissues remains unknown, and this mechanism is addressed in the current study. First, we found that intrOv continuously shed live organisms in rectal swabs and maintained tissue persistence in mice lacking both ILCs and lymphocytes, but intrOv no longer shed live organisms and only maintained tissue persistence in mice deficient in lymphocytes alone, validating the role of innate lymphoid cells in preventing rectal shedding and suggesting a critical role of lymphocytes in blocking intrOv persistence. Second, we have identified the responsible lymphocyte subsets as iNKTs, as mice deficient in CD1d, β2m, or Traj18 allowed intrOv to persist. CD1d, in association with β2m, is required for presenting antigen to iNKTs that use Traj18 as a critical component of their TCRs. Third, we found that IFNγ-producing iNKTs or NKT1 cells are sufficient to clear intrOv persistence: IntrOv induced IFNγ^+^iNKTs in the large intestine, and adoptive transfer of iNKTs inhibited intrOv persistence in recipient mice deficient in iNKT cell development or in IFNγ production. Thus, we have demonstrated the necessity and sufficiency of IFNγ^+^iNKTs in preventing intrOv from persisting in the large intestine.

The finding of the inhibition of intrOv persistence by IFNγ^+^iNKTs is essential for fully revealing the mechanisms to improve the safety of the live-attenuated oral vaccine intrOv. The absence of live intrOv shedding in the rectal swabs and the lack of intrOv persistence in the GI tract tissues of wild-type mice are likely due to the functions of IFNγ^+^ILC3s (37, 38) and IFNγ^+^iNKTs (current study), respectively. The safety of intrOv in the GI tract likely depends on the normal function of IFNγ^+^ILC3s and IFNγ^+^iNKTs in subjects who are orally vaccinated with intrOv. However, individuals with diseases requiring therapies that may cause immunosuppression may reduce their levels of IFNγ^+^ILC3s and/or IFNγ^+^iNKTs, leading to shedding and/or persistence of intrOv. To ensure the safety of intrOv in these immunocompromised subjects, strategies may be used to boost IFNγ^+^ILC3 and/or IFNγ^+^iNKT responses following intrOv inoculation.

Besides inhibiting intrOv in the GI tract (to ensure its safety as discussed above), the intrOv-induced IFNγ^+^ILC3 and IFNγ^+^iNKT responses may also promote intrOv induction of transmucosal protection in the genital tract. Efforts are ongoing to test the hypothesis that IFNγ^+^ILC3s and IFNγ^+^iNKTs may create a tissue environment that promotes the induction of an IFNγ-dominant adaptive immune response. Since IFNγ is the most effective cytokine for inhibiting chlamydial infection, promoting intrOv induction of IFNγ-dominant adaptive immune response in the genital tract should significantly improve the transmucosal protection efficacy of the oral vaccine intrOv. Improving the protection efficacy of intrOv in the genital tract is especially important when intrOv is used to immunize immunocompromised subjects who may have reduced levels of IFNγ^+^ILC3s and/or IFNγ^+^iNKTs. Since IFNγ^+^ILC3s and IFNγ^+^iNKTs are components of innate immunity, it is possible to develop an adjuvant strategy to enhance their responses during intrOv oral immunization. Clearly, strategies to promote IFNγ^+^ILC3 and IFNγ^+^iNKT responses during intrOv immunization may improve both the safety and efficacy of intrOv, especially for individuals with immunocompromised conditions. Given the conservation of innate immunity components between mice and humans, approaches learnt from mouse models may be applicable to humans.

Since IFNγ^+^ILC3s are required for blocking the shedding of live intrOv (37, 38), while IFNγ^+^iNKTs are for preventing intrOv persistence (current study), we might have revealed a division of labor between IFNγ^+^ILC3s and IFNγ^+^iNKTs in regulating chlamydial interactions with host mucosal tissues, potentially by delivering the same effector molecule IFNγ to different target cells laden with chlamydial organisms, although direct evidence for supporting this hypothesis is to be obtained. From a microbial-host interaction perspective, the microbial goal is to pass its genetic material from generation to generation, which can be achieved by persisting in infected host tissue for long periods and/or by spreading to new hosts. The persistent microbe can also serve as a reservoir, repeatedly promoting microbial spreading. A prototypical example is the herpes simplex virus (HSV), which can both maintain latency and cause productive infection (57). HSV persists in the dorsal root ganglia (to prolong colonization in the infected host) and productively infects mucocutaneous cells (to produce infectious progenies for spreading to new hosts). Likely, the obligate intracellular Chlamydia may also infect various cell types in distinct histological regions of the large intestine, such as colonic epithelial cells distributed along villi (8). These epithelial cells exhibit distinct properties, ranging from stem-like cells at the bottom of a crypt to mature epithelial cells ready to shed at the tip of a villus. Distinct colonic epithelial cells may have different susceptibilities to chlamydial infection, allowing intracellular growth for varying periods and to varying extents, with villus-bottom epithelial cells supporting persistence while tip epithelial cells release infectious progeny into the colon lumen, enabling spread to new hosts. We hypothesize that live intrOv detected in a rectal swab is more likely released from the villus-tip epithelial cells, whereas the intrOv recovered from tissue homogenates may mainly come from villus-bottom epithelial cells. Furthermore, *C. muridarum* is known to infect myeloid cells, including DCs (58). Thus, intrOv organisms recovered from the large intestinal tissues may also originate from infected DCs in the lamina propria. Although testing the above hypotheses is still underway, we can speculate that the blockade of live intrOv shedding by IFNγ^+^ILC3s may depend on the ability of ILC3s to deliver IFNγ to the villus-tip epithelial cells, while the prevention of intrOv persistence by IFNγ^+^iNKTs may require iNKTs to deliver IFNγ to the villus-bottom stem-like epithelial cells or DCs in the lamina propria. Thus, the division of labor between IFNγ^+^ILC3s and IFNγ^+^iNKTs is likely driven by chlamydial interactions with distinct colonic cells. This hypothesis is consistent with the concept that intestinal epithelial cells may mediate and dictate interactions between the lumenal microbiota and immune cells (59, 60). It will be interesting to test whether IFNγ^+^ILC3s selectively interact with the villus-tip epithelial cells while IFNγ^+^iNKTs interact with the villus-bottom epithelial cells or lamina propria myeloid cells. Furthermore, it is also worthwhile to determine whether the division of labor between IFNγ^+^ILC3s and IFNγ^+^iNKTs reflects an existing physiological interaction pattern or is induced by chlamydial colonization.

The finding that different IFNγ-producing cells are able to deliver IFNγ to distinct target cells may provide novel insights into innate immune mechanisms that regulate interactions between the microbiota and host mucosal tissues. As wild-type *C. muridarum* continuously sheds live organisms in rectal swabs and persists in the large intestine for long periods without causing significant pathology, *C. muridarum* has been proposed to be a commensal species in the large intestine. Although the division of labor between IFNγ^+^ILC3s and IFNγ^+^iNKTs was discovered using the attenuated *C. muridarum* mutant intrOv, it is likely that both wild-type and mutant *C. muridarum* can induce similar IFNγ^+^ILC3s and IFNγ^+^iNKTs responses in the large intestine. The difference may be that wild-type *C. muridarum* is refractory, while mutant *C. muridarum* intrOv is susceptible to the same set of immune responses. Thus, our findings may have revealed a novel mucosal mechanism that regulates interactions between the microbiota and host mucosal tissues. This hypothesis is consistent with the observation that the microbiota-maintained basal levels of IFNγ in the intestine can be contributed by either IFNγ^+^ILC3s or IFNγ^+^iNKTs (39, 61, 62). The discovery that different subsets of innate lymphoid cells/lymphocytes have specialized into related but distinct functions in the GI tract may also help guide experimental design to elucidate the immunopathogenic mechanisms induced by chlamydia in the genital tract.

We are aware of the limitations of the current study. First, inhibition of intrOv in the large intestine may involve many layers of host responses. Although our previous experiments and current study have mainly focused on the roles of IFNγ^+^ILC3s and IFNγ^+^iNKTs, there may be additional host responses that contribute to the clearance of intrOv in wild-type mice. For example, β2m^-^/^-^ mice developed more extensive shedding than CD1d^-^/^-^, which may suggest a role of CD8^+^ T cells in reducing chlamydial shedding. In a separate study, we are using an adoptive transfer approach to evaluate the contribution of CD8^+^ T cells to shedding clearance in Rag2-/- recipient mice and characterizing the nature of the responsible CD8+ T cells. Second, the concept of IFNγ delivery by IFNγ^+^ILC3s and IFNγ^+^iNKTs to distinct chlamydia-laden cells in the large intestine is established based on functional data. Direct evidence to demonstrate the division of labor between IFNγ^+^ILC3s and IFNγ^+^iNKTs is still lacking. For example, in our adoptive transfer experiment, the question on how donor cell-mediated inhibition of intrOv in the large intestine remains to be addressed. In addition, although the transferred donor cells significantly reduced the intrOv yields in recipient mice, the intrOv organisms were not completely cleared, which may be due to an insufficient number of donor cells or a lack of cooperating cells. These issues need to be addressed to fully understand how the donor lymphoid cells inhibit intrOv in the large intestine. Finally, the current study is based solely on the convenient intracolonic inoculation model, which bypasses exposure to the upper GI tract and small intestinal transit. This is different from the pathways of the actual oral vaccination. Having learned the division of labor between different lymphoid subsets from the colonic model, we are comparing immune response profiles induced via oral versus intracolonic inoculation with intrOv in a separate study. It will also be interesting to determine whether oral vaccination can induce IFNγ^+^iNKT response and whether the intrOv-induced IFNγ^+^iNKT response is necessary for oral intrOv induction of transmucosal protection in the genital tract.

## Data Availability

The original contributions presented in the study are included in the article/**Supplementary Material**. Further inquiries can be directed to the corresponding author.

## Supplementary material

Supplementary Figure 1 Scheme for donor cell induction, preparation, and injection. Five days after intraperitoneal injection of 2 **μ**g αGalCer in 200 μl PBS per mouse, the spleen and liver were harvested for making single-cell suspensions. Cells were stained with viability stain and antibodies (CD16/32, CD45, TCRβ, αGalCer-CD1d-Tetramer). Live CD45+, NK1.1+, TCRβ+, αGalCer-CD1d-Tetramer+ (iNKTs) or – (Non-iNKTs) were sorted. The sorted cells were injected retro-orbitally into recipient mice 24 hrs. prior to intracolonic challenge with 1x10^7^ intrOv.

Supplementary Figure 2 Single-cell extraction protocol from both the small and large intestine. The small intestine (SI) and large intestine (LI) were harvested from mice and thoroughly minced in a glass Petri dish. The minced tissue samples were digested in a 10 ml solution of collagenase II (1 mg/ml) and DNase (60 μg) by incubating in a shaking incubator for 50 min at 37 ˚C and 180 rpm. After centrifugation at 1000g and 4 ˚C for 6 min, the supernatant was discarded, and 10 mL of EDTA (5mM) +DTT (1mM) solution was used to resuspend the pellet, followed by incubation in a shaking incubator for 20 min at 180 rpm and 37 ˚C. After mixing with 10 ml of FACS buffer, the suspension was filtered through a 100 µM strainer and centrifuged at 1000g and 4 ˚C for 6 min. A 40% Percoll solution was used to resuspend the pellet for Percoll gradient centrifugation. The isolated cells were subjected to flow cytometric analysis after overnight stimulation with a mitogen.

Supplementary Figure 3 Flow cytometric detection of iNKTs from C57BL/6J versus TRAJ18^-/-^ mice. Liver from C57BL/6J mice (Panel **(A)**; with iNKTs) or TRAJ18^-/-^ (**(B)**; lacking iNKT) were harvested, and a single cell suspension was obtained and stained with viability stain and mouse cell surface markers, CD45, αGalactosyl ceramide (αGalCer) loaded CD1d-tetramer, Empty CD1d-tetramer (not loaded with αGalCer), and CD3ε. Note that only the αGalCer-loaded CD1d tetramer identified iNKT cells in the liver of C57BL/6J, but not TRAJ18^-/-^.

Supplementary Figure 4 Gating strategy for sorting αβiNKTs and αβMAITs. Five days after C57BL/6J mice were injected intraperitoneally with 200µl of αGalactosyl ceramide (αGalCer, 10µg/ml), mice were sacrificed for harvesting spleen and liver. Single cell suspension was obtained and stained with viability stain and mouse cell surface markers αGalCer-loaded CD1d-tetramer, CD45, NK1.1, TCRβ. αβiNKTs (Nk1.1+TCRβ+αGalCer-loaded CD1d-Tetramer+) or αβMAITs (Nk1.1+TCRβ+αGalCer-loaded CD1d-Tetramer-) were sorted as shown in the representative flow cytometry gating diagram.

Supplementary Figure 5 Gating strategy for the identification of αβiNKTs. Cells isolated from the large intestine, small intestine, and liver tissue were stained for viability and mouse cell surface markers, including αGalactosyl ceramide (αGalCer)-loaded CD1d-tetramer, CD45, NK1.1, TCRβ, and IFNγ. (Related to **Figures 5A–D**).
