# Supplementary figures and images for: IFNγ-producing iNKTs restrict a live-attenuated chlamydia oral vaccine in the large intestine
Source: Front Immunol. 2026 Jun 1;17:1851941. doi: 10.3389/fimmu.2026.1851941 (PMC13284539; doi:10.3389/fimmu.2026.1851941)

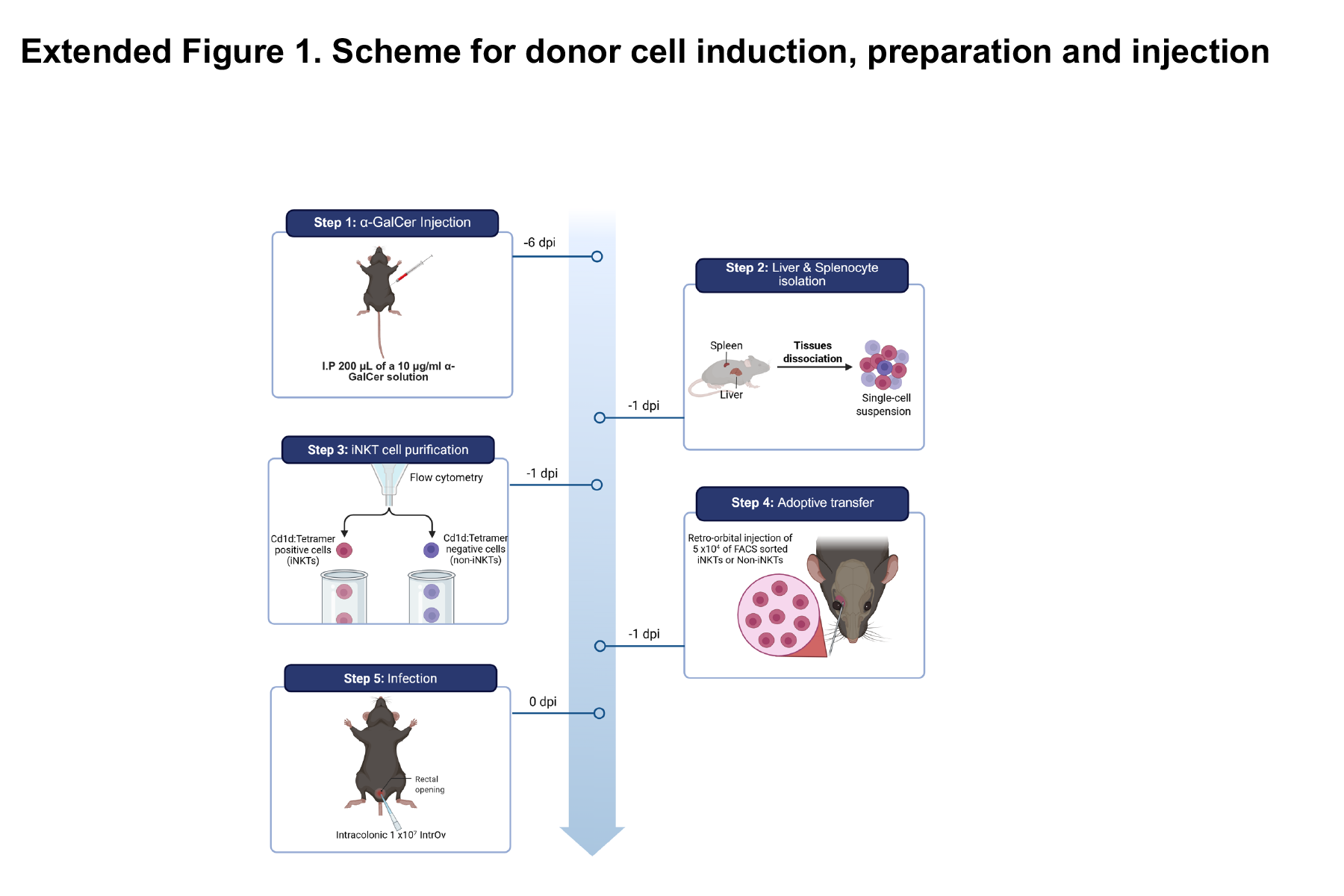

Supplement: Supplementary Figure 1 — Scheme for donor cell induction, preparation, and injection. Five days after intraperitoneal injection of 2 μg αGalCer in 200 μl PBS per mouse, the spleen and liver were harvested for making single-cell suspensions. Cells were stained with viability stain and antibodies (CD16/32, CD45, TCRβ, αGalCer-CD1d-Tetramer). Live CD45+, NK1.1+, TCRβ+, αGalCer-CD1d-Tetramer+ (iNKTs) or – (Non-iNKTs) were sorted. The sorted cells were injected retro-orbitally into recipient mice 24 hrs. prior to intracolonic challenge with 1x10^7^ intrOv. [file Image1.tif]

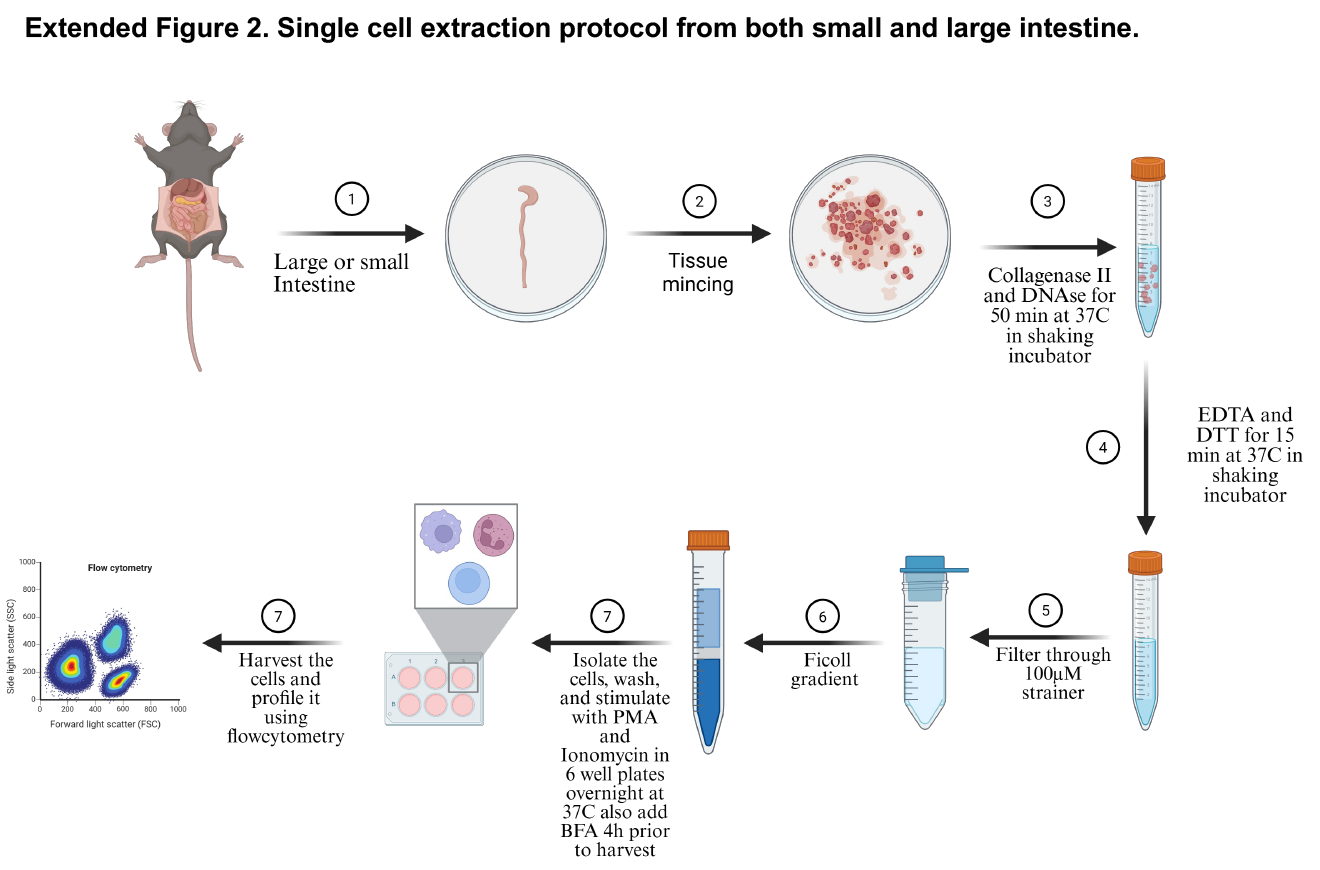

Supplement: Supplementary Figure 2 — Single-cell extraction protocol from both the small and large intestine. The small intestine (SI) and large intestine (LI) were harvested from mice and thoroughly minced in a glass Petri dish. The minced tissue samples were digested in a 10 ml solution of collagenase II (1 mg/ml) and DNase (60 μg) by incubating in a shaking incubator for 50 min at 37 ˚C and 180 rpm. After centrifugation at 1000g and 4 ˚C for 6 min, the supernatant was discarded, and 10 mL of EDTA (5mM) +DTT (1mM) solution was used to resuspend the pellet, followed by incubation in a shaking incubator for 20 min at 180 rpm and 37 ˚C. After mixing with 10 ml of FACS buffer, the suspension was filtered through a 100 µM strainer and centrifuged at 1000g and 4 ˚C for 6 min. A 40% Percoll solution was used to resuspend the pellet for Percoll gradient centrifugation. The isolated cells were subjected to flow cytometric analysis after overnight stimulation with a mitogen. [file Image2.tif]

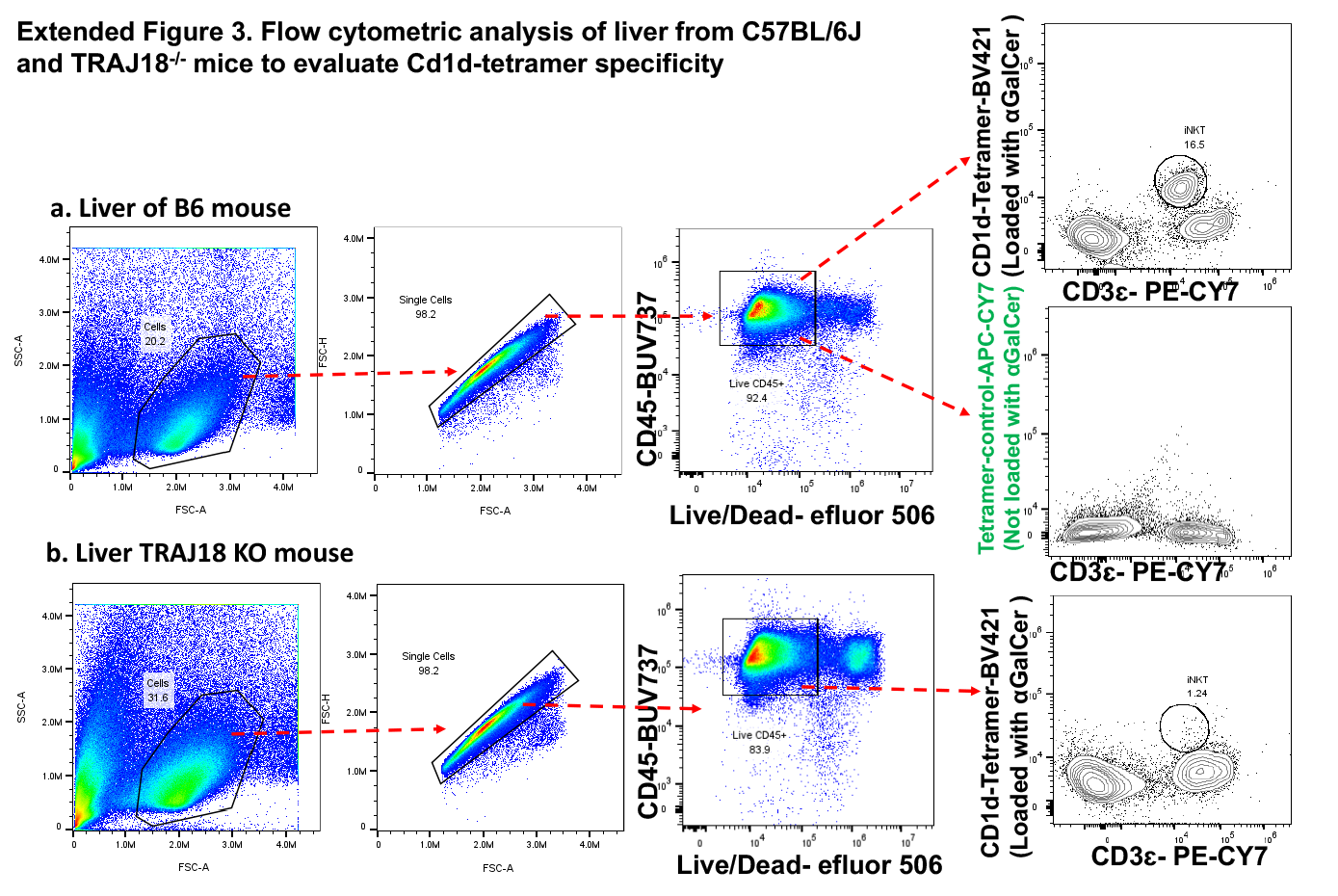

Supplement: Supplementary Figure 3 — Flow cytometric detection of iNKTs from C57BL/6J versus TRAJ18-/- mice. Liver from C57BL/6J mice (Panel (A); with iNKTs) or TRAJ18-/- ((B); lacking iNKT) were harvested, and a single cell suspension was obtained and stained with viability stain and mouse cell surface markers, CD45, αGalactosyl ceramide (αGalCer) loaded CD1d-tetramer, Empty CD1d-tetramer (not loaded with αGalCer), and CD3ε. Note that only the αGalCer-loaded CD1d tetramer identified iNKT cells in the liver of C57BL/6J, but not TRAJ18-/-. [file Image3.tif]

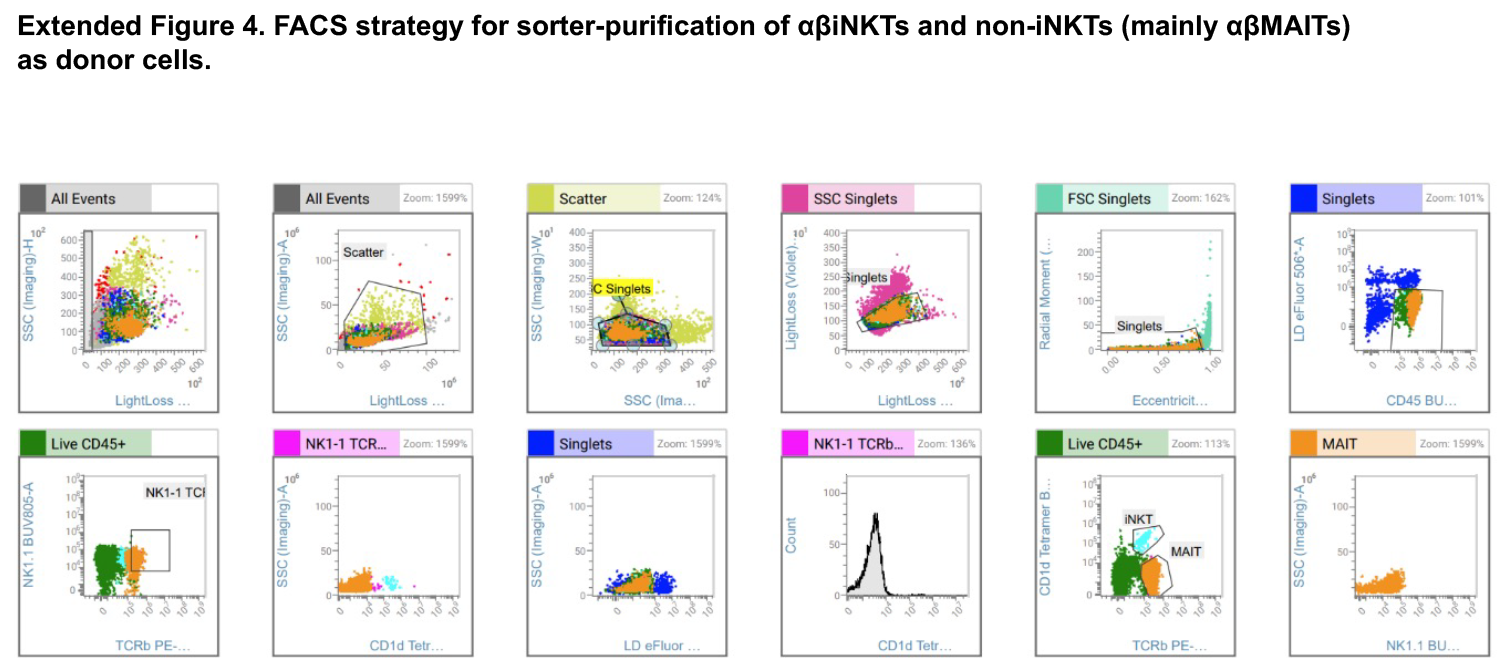

Supplement: Supplementary Figure 4 — Gating strategy for sorting αβiNKTs and αβMAITs. Five days after C57BL/6J mice were injected intraperitoneally with 200µl of αGalactosyl ceramide (αGalCer, 10µg/ml), mice were sacrificed for harvesting spleen and liver. Single cell suspension was obtained and stained with viability stain and mouse cell surface markers αGalCer-loaded CD1d-tetramer, CD45, NK1.1, TCRβ. αβiNKTs (Nk1.1+TCRβ+αGalCer-loaded CD1d-Tetramer+) or αβMAITs (Nk1.1+TCRβ+αGalCer-loaded CD1d-Tetramer-) were sorted as shown in the representative flow cytometry gating diagram. [file Image4.tif]

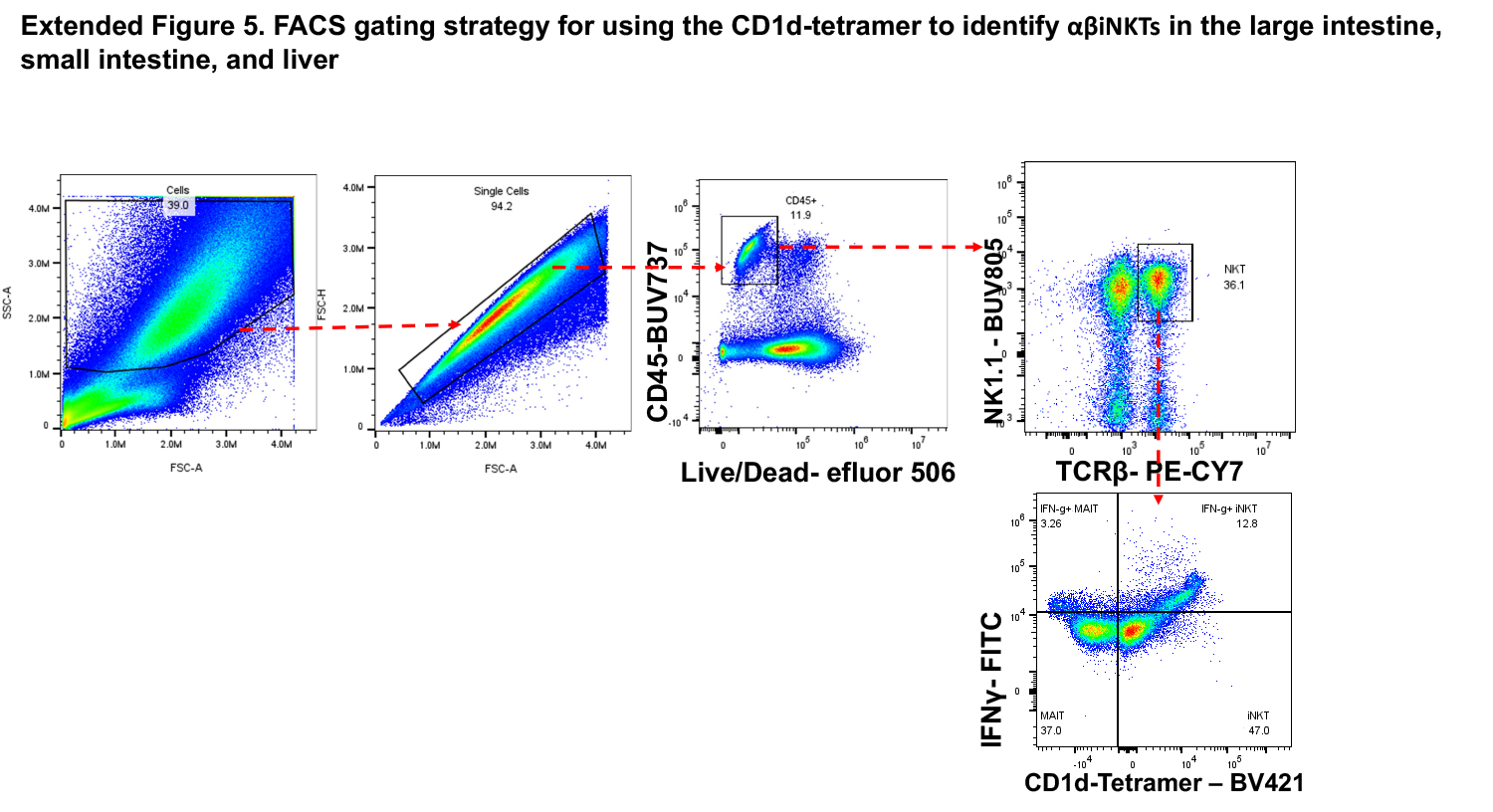

Supplement: Supplementary Figure 5 — Gating strategy for the identification of αβiNKTs. Cells isolated from the large intestine, small intestine, and liver tissue were stained for viability and mouse cell surface markers, including αGalactosyl ceramide (αGalCer)-loaded CD1d-tetramer, CD45, NK1.1, TCRβ, and IFNγ. (Related to Figures 5A–D). [file Image5.tif]
